# Revisiting Myositis Ossificans: A Comprehensive Stage-by-Stage Imaging Review

**DOI:** 10.3390/muscles5020027

**Published:** 2026-04-14

**Authors:** Consolato Gullì, Giuseppe Ferrara, Emanuele Ferravante, Roberto Calbi, Mario Di Diego, Davide Parisi, Daniele Perla, Tommaso Villa, Luigi Natale

**Affiliations:** 1 Advanced Radiology Center, Department of Diagnostic Imaging and Radiation Oncology, Fondazione Policlinico Universitario A. Gemelli IRCCS, 00136 Rome, Italy; mario.didiego@guest.policlinicogemelli.it (M.D.D.); daniele.perla@guest.policlinicogemelli.it (D.P.); luigi.natale@unicatt.it (L.N.); 2 Section of Radiology, Department of Radiological and Hematological Sciences, Università Cattolica del Sacro Cuore, 00136 Rome, Italy; giuseppe.ferrara07@icatt.it (G.F.); emanuele.ferravante01@icatt.it (E.F.); davide.parisi01@icatt.it (D.P.); t.ommasovilla@outlook.it (T.V.); 3 Department of Radiology, Ospedale Regionale Miulli, 70021 Bari, Italy; r.calbi@miulli.it

**Keywords:** myositis ossificans, heterotopic ossification, MRI, CT, ultrasound, differential diagnosis, zonal phenomenon, soft tissue tumors, mimics

## Abstract

Myositis ossificans (MO) is a benign, self-limiting heterotopic ossification process that typically develops within soft tissues following trauma, although non-traumatic forms have also been described. Despite its benign nature, MO frequently represents a diagnostic challenge, particularly in its early stages when imaging findings may mimic aggressive soft-tissue tumors, leading to unnecessary biopsies or surgical interventions. This narrative review provides an updated overview of the classification, pathophysiology, and imaging features of myositis ossificans, with a specific focus on the time-dependent evolution of radiologic appearances across different imaging modalities. Radiologic findings are discussed according to disease stage, highlighting key diagnostic clues such as the zonal phenomenon and peripheral maturation pattern. In addition, the main entities included in the differential diagnosis are reviewed, with particular emphasis on imaging features that help distinguish myositis ossificans from soft-tissue sarcomas and other calcified or ossified lesions. Finally, current management strategies and the role of imaging in patient follow-up are summarized. A thorough understanding of the evolving imaging spectrum of myositis ossificans is essential for radiologists and clinicians to achieve an accurate diagnosis, guide appropriate management, and avoid overtreatment.

## 1. Introduction

Myositis ossificans (MO) is a condition characterised by the formation of heterotopic, non-neoplastic bone within soft tissues, including not only skeletal muscle but also tendons, ligaments, and fasciae [1,2]. Although the present review focuses on human pathology, similar heterotopic ossification processes have also been described in veterinary medicine. Fundamentally, MO is categorised into two distinct types: the non-hereditary (acquired) forms and the hereditary form [1,2]. This review focuses on the more common acquired forms, which are self-limiting and often post-traumatic, although an inciting event is often inapparent [1,2,3]. The term “myositis” is regarded as a misnomer; as Kransdorf et al. noted [3], no primary inflammation of skeletal muscle is associated with the process. This entity presents a significant diagnostic challenge because its imaging appearance, especially during its early development, can closely mimic the features of a malignant tumor [1,2,3]. From a radiologic perspective, myositis ossificans should be regarded as a dynamic process, in which imaging appearances evolve in a time-dependent and stage-specific manner, making awareness of disease chronology essential for correct interpretation. The present review first summarizes the classification, epidemiology, and histopathological basis of myositis ossificans. Subsequently, the radiological evolution of the disease is discussed using a stage-by-stage approach, followed by the main differential diagnoses and current management strategies.

## 2. Methods

This narrative review was conducted through a structured literature search in PubMed, Scopus, and Web of Science. The search included articles published in English using the following keywords: “myositis ossificans”, “heterotopic ossification”, “soft-tissue calcification”, “MRI”, “CT”, and “ultrasound”. Relevant studies focusing on the pathophysiology, imaging findings, and differential diagnosis of myositis ossificans were selected. Both original articles and review papers were considered when they provided relevant radiological correlations. The selection of studies was based on their relevance to the imaging-oriented scope of the present narrative review. Articles not addressing imaging findings or radiological correlations of myositis ossificans were excluded. The literature search included articles published up to January 2026.

## 3. Classification

MO is fundamentally categorized into non-hereditary and hereditary types.

Early classification schemes attempted to categorize the entity based on aetiology or temporal progression:

- Lewis’s classification organized MO into traumatic, non-traumatic, and neurotic types [4];
- Nobles’s classification suggested categories including MOP, circumscribed traumatic, and circumscribed without a history of trauma [5].

However, recently recognized clinical and radiological subtypes of myositis ossificans, as illustrated by Tyler and colleagues, comprise three principal entities: Post-traumatic MO (PTMO), Nontraumatic/Pseudomalignant MO, and Fibrodysplasia ossificans progressiva (FOP) [1]:

- PTMO represents the most common nonhereditary form. It is a benign, usually solitary reactive form of heterotopic ossification that, while typically linked to trauma, may occur without a confirmed history in up to 40% of cases.
- Nontraumatic/Pseudomalignant MO includes Neurogenic Heterotopic Ossification (NHO), often complicating spinal cord injury (occurring below the level of the spinal cord injury) and heterotopic ossification following thermal burns [1].
- FOP, previously known as myositis ossificans progressiva (MOP), is an extremely rare hereditary disorder characterized by progressive heterotopic ossification of connective tissues [1,6].

## 4. Epidemiology and Risk Factors

PTMO accounts for 60–75% of all cases of MO [1,7]. Generally, this condition can occur at any age, but it is predominantly observed in adolescents and young adults [7]. More than half of the cases occur in the second and third decades of life, with few reported cases involving children under the age of 10 [1,7]. A recent study reported an average age of 36 years, with a wide range extending from 4 to 84 years [8]. There is a slight male predominance, which is largely attributed to higher levels of participation in physical activities and contact sports [1,2].

It typically affects large and active muscle groups [1]. Statistical analysis reveals a strong predilection for the lower limbs (approximately 73%) compared to the upper limbs (26%) [8]. The quadriceps femoris is the most frequently affected muscle group [8], with specific involvement of the vastus intermedius and vastus lateralis being particularly common [1,8]. In the upper limb, the brachialis and triceps muscles are the most common sites [1,7,8]. Generally, the anterior muscle groups are involved more frequently than the posterior, and proximal regions of an extremity are affected more often than distal parts [1,7]. Although rare, lesions can also occur in the hand and wrist, involving ligaments such as the scapholunate ligament [9].

The most significant risk factor is a history of trauma, which may consist of a single severe blow or repetitive minor trauma [2,7]. The likelihood of developing PTMO correlates with the severity of the initial injury [1]. Specific occupational or athletic micro-traumas have led to historically named variants, such as “rider’s bone” in the adductor muscles of horseback riders and “shooter’s bone” in the deltoid of rifle shooters [2]. Furthermore, modern activities such as yoga have been recognised as potential sources of repetitive microtrauma, because weight-bearing poses can generate significant mechanical strain across joints, leading to ossification even in ligamentous structures [9].

Bleeding disorders represent another significant risk factor, a finding that supports the hypothesis that the condition is intrinsically linked to hematoma formation. Moreover, a decreased range of motion (ROM) following injury is considered a notable predictor for the development of the disease; indeed, it has been observed that specific limitations, such as knee flexion of less than 120 degrees following a thigh injury, correlate with an increased risk of developing MO [2].

Despite its name, a clear history of trauma is absent in approximately 40% of cases [1,7]. In these instances, the inciting event may have been a minor muscle injury unrecognized by the patient [2].

MO is also associated with conditions causing prolonged immobility, such as paraplegia, spinal cord injuries (NHO), tetanus, and polio, and is observed in approximately 2% to 3% of patients with thermal burns [7].

## 5. Histopathological Staging and Clinical Presentation

MO results from dysregulated mesenchymal stem cell activation following tissue trauma, mediated by osteoinductive cytokines that promote differentiation into chondrocytes and osteoblasts and subsequent endochondral ossification within extraskeletal tissues [2]. This pathophysiological mechanism is common to all acquired forms regardless of aetiology [2]. These histopathological changes directly correspond to the characteristic imaging findings observed during disease evolution. In particular, the progressive peripheral maturation of the lesion reflects the development of lamellar bone at the periphery, which explains the typical radiologic “zonal phenomenon.” Recent studies suggest that heterotopic bone formation in myositis ossificans involves activation of osteogenic signaling pathways, particularly bone morphogenetic proteins (BMP-2 and BMP-4), which promote the differentiation of mesenchymal progenitor cells into osteoblast lineage cells [2,10]. The newly formed bone undergoes a maturation process similar to endochondral ossification, involving osteoblasts, osteocytes, and osteoclast-mediated remodeling [2]. Molecular pathways such as the RANK/RANKL/OPG system, together with extracellular matrix proteins including osteocalcin and osteopontin, as well as growth factors such as TGF-β, have also been implicated in the regulation of bone formation and remodeling in heterotopic ossification [2,10]. The clinical, radiological, and histopathological evolution proceeds through three distinct phases:

The early phase (0–4 weeks) comprises an acute inflammatory stage (0–1 week) and a subacute stage (weeks 1–4). During the acute phase, there is a vascular response, leukocyte infiltration, and release of osteoinductive cytokines including bone morphogenetic proteins (BMPs 2 and 4) and transforming growth factor-beta (TGF-β) [1,2]; histologically, mesenchymal cells secrete a myxoid matrix, and fibroblasts exhibit numerous mitoses, creating a pseudo-fibrosarcomatous appearance [10]. During the subacute stage, fibroblasts differentiate into osteoblasts and secrete an osteoid matrix at the periphery of the myxoid zone, giving the lesion a pseudo-osteosarcomatous appearance [10]. At this stage, MO presents with focal pain, soft tissue swelling, and erythema following a trauma [1,2,7]. Patients report persistent muscle pain extending beyond typical muscle strain, and decreased ROM represents the most common initial clinical manifestation [1,2]. Fever may be present, and the erythrocyte sedimentation rate is typically elevated [1,2].

During the intermediate phase (4–8 weeks), calcifications become increasingly coarse and dense at the periphery, while the central zone retains a relatively soft tissue consistency similar to normal muscle; by 6 to 8 weeks, a well-defined peripheral cortex becomes evident [1,2]. This evolution represents the characteristic “zone phenomenon”, characterized by a distinct pattern of zonal maturation with concentric organization where tissue maturation progresses from the centre to the periphery and is one of the defining features of MO [1,2,10]. Therefore, this architecture comprises three distinct zones: Zone 1 (Central) consists of immature, non-ossified fibroblastic tissue with necrotic debris; Zone 2 (Intermediate) demonstrates osteoid and immature bone formation; and Zone 3 (Peripheral) displays well-organized mature lamellar bone [8,10,11]. As the lesion evolves, a palpable circumscribed soft-tissue mass becomes clinically evident, and joint stiffness progresses [2]. Pain typically results from mechanical irritation of surrounding tissues; however, expansion of the lesion may compress adjacent neurovascular structures, causing paresthesias, weakness, or lymphedema [2].

The mature phase (>8 weeks) is characterized by pronounced peripheral bone formation and represents the culmination of lesion maturation [2]. Mature lesions often display faint internal calcification or trabeculation, with a denser calcified periphery, and by 6 months, the calcified mass characteristically shrinks in size with improved separation from underlying bone [1]. As the lesion consolidates, it is usually accompanied by progressive symptom improvement and functional recovery [2]. Patients presenting late in the disease course may have minimal or absent symptomatology [2,7].

## 6. Radiological Staging

The heterogeneous composition of musculoskeletal tissues requires a multimodality imaging approach. US demonstrates early soft tissue changes before calcifications become radiographically evident [1,2]. Radiography permits visualization of calcifications and enables initial assessment of the characteristic zonal pattern [1,2,7]. CT refines the evaluation of zonal maturation and provides superior cross-sectional delineation of the lesion in relation to adjacent soft tissues and bone [1,2]. MRI is the gold standard for soft tissue evaluation and can identify early lesions preceding radiographic changes, though calcifications are poorly depicted due to short T2 relaxation times, potentially giving the false impression of an immature lesion; consequently, MRI must be interpreted in conjunction with radiographs or CT to avoid diagnostic confusion [2]. Bone scintigraphy may detect increased metabolic activity in early stages of myositis ossificans before mineralization becomes visible on radiographs. Increased tracer uptake is typically observed during the early inflammatory phase and may help assess lesion maturity before surgical excision. However, due to the widespread availability and superior anatomical resolution of CT and MRI, bone scintigraphy is currently less frequently used in routine clinical practice.

### 6.1. Early Phase

**Figure 1:**
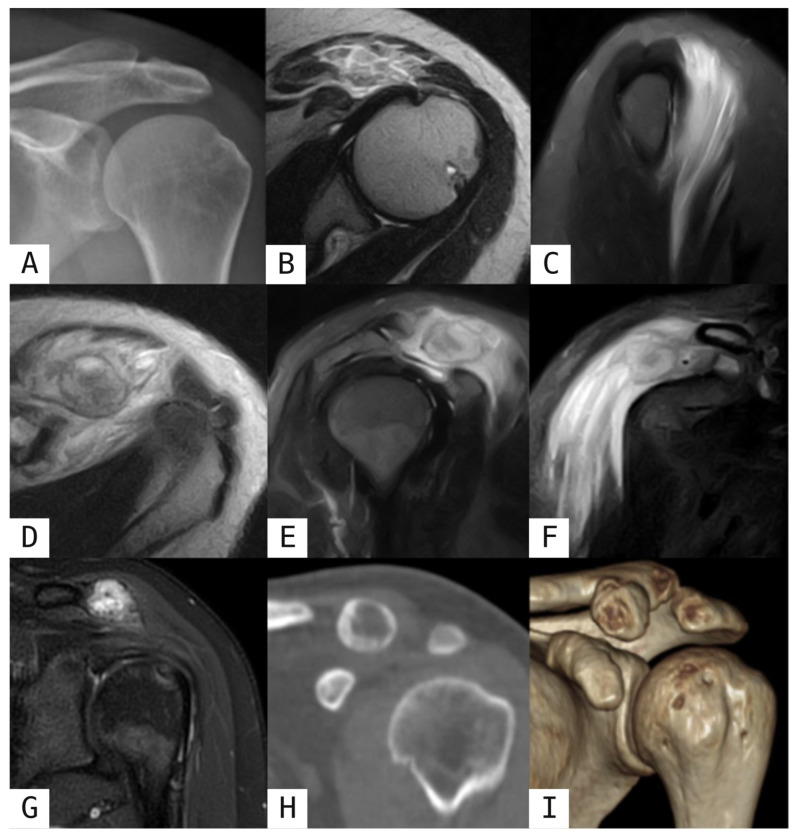
Myositis ossificans in the left deltoid muscles. A 28-year-old woman presented with two weeks of non-traumatic left shoulder pain. X-rays were unremarkable (**A**). MRI axial T2-weighted (**B**) and sagittal STIR (**C**) images showed diffuse oedema and enlargement of the anterior deltoid with the checkerboard pattern on the axial plane and the striated pattern on the sagittal plane. After 5 weeks, follow-up MRI axial T2-weighted (**D**), sagittal STIR (**E**) and coronal STIR sequences (**F**) revealed a 2.5 cm oval lesion in the proximal anterior deltoid, adjacent to the distal clavicle and coracoid process. The lesion had a hypointense peripheral rim and was surrounded by muscle oedema. Six months post-onset, MRI coronal PD fat-sat (FS) sequence (**G**) showed muscle oedema resolution and multiplanar CT coronal reconstruction (**H**) and 3D rendering (**I**) revealed the lesion’s peripheral ossification.

Early MO manifests on US as heterogeneous hypoechoic soft-tissue mass with internal echogenic foci reflecting incipient mineralization [1,2]. The characteristic zonal architecture becomes evident before ossification appears on other imaging modalities, comprising an outer thin peripheral hypoechoic zone with Doppler-demonstrated hyperaemia in the surrounding muscle enclosing a middle hyperechoic zone representing early calcification, and an inner hypoechoic component corresponding to immature fibroblastic stroma [1,2,10]. Plain radiographs may initially appear normal or demonstrate soft tissue swelling with possible localized periosteal reaction reflecting underlying subperiosteal haemorrhage [1,2,7]. Within 7–14 days, localized soft-tissue opacity becomes apparent, and by the third to fourth week, flocculent calcifications develop within the soft-tissue mass as osteoid mineralizes (Figure 1A) [1,2,7]. CT in early stages reveals soft-tissue swelling or low-attenuation masses typically without calcifications, though faint peripheral calcification may begin appearing with evident periosteal changes [1,2,7]. MRI demonstrates a focal mass-like area with isointense to slightly hyperintense T1-weighted signal compared to muscle, or slightly hyperintense signal from methaemoglobin if hematoma is present. Fluid-sensitive sequences are more informative, showing marked diffuse hyperintensity with characteristic perilesional muscle oedema extending along fiber planes. Consequently, two diagnostic patterns of oedema distribution along fascicular architecture are recognized based on the imaging plane: the striatal pattern, on the longitudinal plane, presents with alternating hyperintense and hypointense bands parallel to the muscle long axis; the checkerboard pattern, seen on transverse images, is characterized by a grid-like appearance. The preservation of muscle fascicles within the lesion, which remain identifiable despite infiltration oedema, is a typical imaging finding (Figure 1B,C) [2,8,12]. Post-gadolinium images show characteristic peripheral rim-shaped enhancement respecting the striatal pattern, corresponding to hypervascular osteoid matrix, though diffuse enhancement may occur in very early phases [2,8,12].

### 6.2. Intermediate Phase

**Figure 2:**
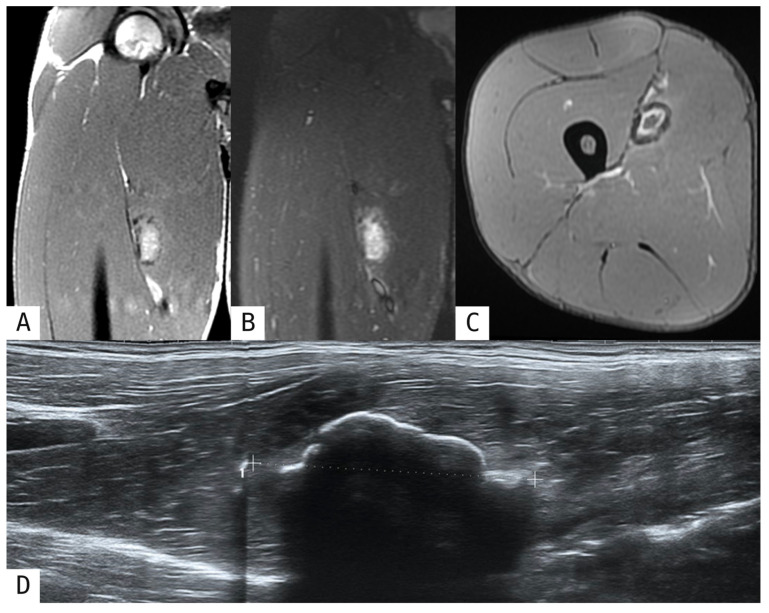
Post-traumatic myositis ossificans in the adductor longus. A 31-year-old soccer player presented with moderate pain in the right thigh two months after feeling a muscle strain while sprinting to get the ball. MRI coronal T1-weighted (**A**), coronal STIR (**B**), and axial MERGE (**C**) images showed a well-defined oval lesion with peripheral hypointensity and faint perilesional oedema in the adductor longus. Ultrasound examination was performed, with a longitudinal scan (**D**) confirming a mass with a peripheral calcified margin, consistent with the intermediate phase of myositis ossificans. The thin white dashed line indicates the maximum lesion diameter measured on ultrasound.

**Figure 3:**
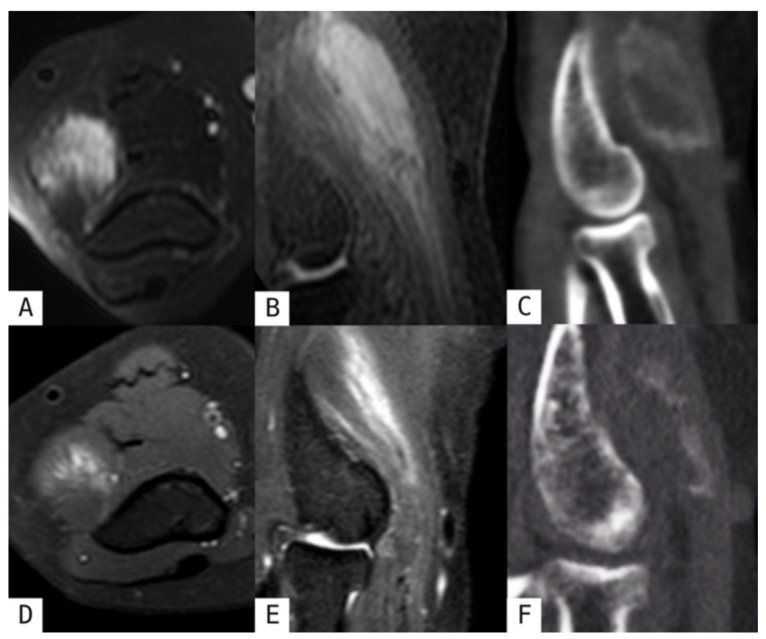
Elbow myositis ossificans before and after corticosteroid treatment. A 65-year-old woman presented with two months of post-traumatic right elbow pain. Axial T2-weighted FS (**A**) and sagittal STIR (**B**) sequences demonstrated marked diffuse oedema (“striated pattern”) with enlargement of the distal brachioradialis muscle and its myotendinous junction. Multiplanar CT sagittal reconstruction (**C**) revealed peripheral eccentric calcifications. One month after corticosteroid therapy, follow-up axial PD (**D**) and sagittal STIR (**E**) sequences revealed reduced oedema and lesion size, while sagittal 2D MPR reconstruction CT (**F**) showed regression of the peripheral calcifications.

Progressive peripheral calcification becomes increasingly evident on US, with the lesion developing peripheral sheet-like lamellar ossification which leads to increased echogenicity and posterior acoustic shadowing [1,7] (Figure 2D). Moreover, calcifications on radiography and CT become progressively more peripherally distributed and coarser in appearance. The pathognomonic zone phenomenon, characterized by peripheral calcific densification with a relatively radiolucent central zone, emerges as the most important diagnostic feature indicating benign pathology. By 6–8 weeks, organized peripheral ossification with a well-defined cortex develops, and ossifications characteristically align parallel to the long axis of the affected muscle fibers [1,2,7] (Figure 3C). On MRI, T1-weighted sequences demonstrate central hyperintensity from fatty metaplasia and marrow formation, while peripheral hypointensity develops from mineralization. T2-weighted and fluid-sensitive imaging shows a peripheral hypointense rim reflecting advancing ossification, with progressive reduction in perilesional oedema [2,8,12] (Figure 1D–F, Figure 2A–C and Figure 3A,B).

### 6.3. Mature Phase

Mature lesions on US display a highly reflective, completely calcified periphery with markedly increased acoustic shadowing, while shrinkage of the ossified rim, with surface irregularities, and of the affected musculature [1]. Radiographically, mature lesions often display faint internal calcification or trabeculation with a denser calcified periphery or may appear completely dense. By six months, the calcified mass shrinks in size with improved separation from the underlying skeleton; occasionally, mature lesions may be adherent to the underlying bone, mimicking other lesions [1,7]. On CT, mature MO typically demonstrates a characteristic appearance with heavily calcified peripheral ossification that may display a crenated pattern, with the central zone potentially remaining isodense to adjacent muscle, or it may appear uniformly or homogeneously dense [1,2,7] (Figure 1H,I). MRI demonstrates prevalent hypointensity across all sequences, reflecting extensive mineralization and ossified matrix. Central zones show fat signal intensity similar to bone marrow, perilesional oedema is absent, and the peripheral zone demonstrates low signal intensity from mature lamellar bone, as with complete histological maturation [2,8,12] (Figure 1G).

## 7. Differential Diagnosis

The primary radiological challenge lies in accurately differentiating MO from soft tissue malignancies. Not uncommonly, the presentation is atypical, imaging findings are indeterminate, or clinical history is unclear or absent, rendering diagnosis challenging [2,8,13].

### 7.1. Early Phase

In this phase, given the absence of soft-tissue calcifications, accurate guidance in the differential diagnosis of MO relies primarily on a thorough analysis of the clinical and radiological semeiotics of muscle oedema. First, it is essential to determine whether the oedema preserves the muscle fiber architecture (e.g., striated or checkerboard pattern) or whether it disrupts the muscle fascicles, as observed in infiltration oedema or oedema secondary to fiber rupture [14,15]. Second, the localization and distribution of the oedema should be noted as either diffuse or focal within the same muscle. Third, assessment of the affected muscle’s volume is important, distinguishing between hypertrophy, normotrophy, hypotrophy, or atrophy. Fourth, it is necessary to evaluate whether oedema is present in other muscles and whether these muscles share innervation by the same nerve [16].

Muscle oedema can be further categorized into three patterns: diffuse, focal, and multifocal [17]. The diffuse pattern is characterized by bilateral involvement of multiple muscles and muscle groups. The focal pattern is restricted to a single muscle or contiguous region involving adjacent muscles. The multifocal pattern is defined by multiple discrete areas of oedema separated spatially and often appearing patchy or nodular [17]. Each pattern reflects specific pathological aetiologies, summarized in Table 1.

### 7.2. Intermediate Phase

Differentiating MO from extraosseous osteosarcoma is a clinically significant challenge. The most reliable distinguishing feature is calcification pattern; for instance, extraosseous osteosarcoma shows centrifugal calcification (centre towards periphery) with disorganized peripheral calcifications reflecting infiltrative growth. Moreover, osteosarcoma shows irregular, infiltrative margins with soft tissue invasion. On MRI, osteosarcoma demonstrates heterogeneous central or diffuse enhancement with fascicular invasion. On follow-up serial imaging, perilesional oedema persists or increases in osteosarcoma and progressively decreases in MO.

Parosteal osteosarcoma is another important malignant consideration, particularly when MO is located near the bone. This rare lesion originates from the bone surface, either from the periosteum or cortex, and remains firmly attached to bone with periosteal continuity, growing outward into soft tissues, and on radiography shows the radiolucent cleft (string sign) separating the lesion from the bone cortex. Calcifications are immediately evident on initial radiographs, even in very early-stage lesions, reflecting the ossifying nature of the tumor from inception, whereas they are absent in early-phase MO. CT and MRI can clearly show the lesion’s origin from periosteum and cortical surface [2,8,13,18].

**Figure 4:**
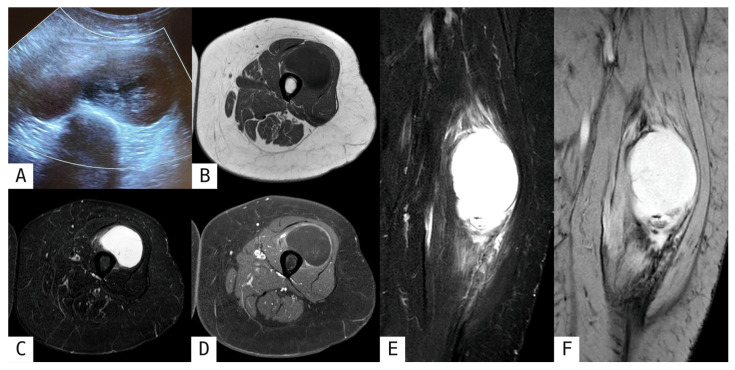
Myxofibrosarcoma of the left thigh. A 67-year-old woman presented with a painless enlarging soft tissue mass in the left thigh one month after a day hike. US transverse scan (**A**) showed a subfascial heterogeneously hypoechoic mass with smooth margins, with no vascular signals on Power-Doppler. MRI axial T1-weighted (**B**), STIR (**C**), T1- weighted FS post-gadolinium (**D**), coronal STIR (**E**), and mFFE (**F**) images showed an oval-shaped lesion in the vastus intermedius with high T2 signal content (**C**,**E**,**F**) and no “blooming” artifact in the gradient-echo sequences (**F**). The lesion had fusiform margins, was surrounded by muscle oedema and compressed the vastus intermedius and rectus femoris muscles. Resection was performed with tumor-free margins. Pathology from an excisional biopsy demonstrated myxofibrosarcoma.

Soft tissue sarcomas may mimic MO in early phases (Figure 4). Undifferentiated pleomorphic sarcoma (UPS, formerly malignant fibrous histiocytoma) is the most common soft tissue sarcoma and shows fascicular invasion, heterogeneous enhancement, and persistently expanding oedema, while MO demonstrates preserved muscle fascicles and peripheral ring-shaped enhancement with progressively decreasing perilesional oedema. UPS rarely calcifies and lacks organized zoning, presents without clear trauma and demonstrates progressive worsening pain, including severe night pain. Serial imaging shows progressive growth and soft-tissue invasion in UPS, versus shrinkage in MO.

Soft tissue infection or abscess classically presents with uniform appearance, T2 hyperintensity, T1 hypointensity, and thick, irregular contrast-enhancing wall, in contradistinction to the smooth peripheral rim enhancement and preserved muscle fascicles of MO. CT with intravenous contrast may demonstrate a fluid collection with an enhancing ring in an abscess, clearly distinguishing it from the complex solid signal patterns of MO. Clinical history, including fever, elevated white blood cell count, and systemic inflammatory markers, differentiates infection from MO, as does the absence of growth on microbial culture [2,8,13,18].

### 7.3. Mature Phase

Osteochondromas are lesions that originate from the bone surface with direct cortical and medullary continuity, in contrast to MO, which is entirely soft tissue-based and separated from bone by the characteristic string sign. MO can be differentiated in the same way with bizarre parosteal osteochondromatous proliferation (also known as Nora lesion), which is a benign lesion that originates from the bone surface with direct osseous continuity to the adjacent cortex.

Melorheostosis is a rare benign sclerotic bone dysplasia, which, in its myositis-like variant which shows mainly soft tissues involvement, can mimic MO; however, its sclerotomal distribution pattern and its close relationship with cortical bone allow differentiation.

**Figure 5:**
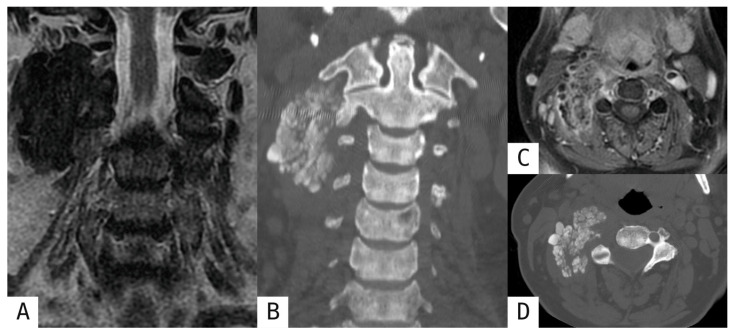
Tumoral calcinosis in the right paravertebral soft tissues. 56-year-old female patient presented with right latero-cervical tenderness. MRI coronal T2-weighted (**A**), axial T1-weighted FS post-gadolinium (**C**) and axial and coronal MPR CT reconstructions (**B**,**D**) showed a well-defined, lobulated, predominantly homogeneous mass, markedly hypointense in T1-weighted and T2-weighted sequences, consistent with multiple amorphous calcifications (better depicted on CT).

Tumoral calcinosis presents with large deposits of amorphous calcifications without organized peripheral zoning, multifocal distribution around joints, and a clinical context of metabolic derangement, including hyperphosphatemia, clearly distinguishing it from MO (Figure 5) [2,8,10,13,18,19]. Some pearls of the aforementioned diagnostic findings for differential diagnosis are summarized in Table 2.

## 8. Treatment

### 8.1. Conservative Management

Non-surgical treatment aims to minimize symptoms and maximize function and is often effective because MO is self-limiting. Initial management includes brief immobilization, rest, ice, compression, and elevation. Cryotherapy reduces intramuscular blood flow, limiting hematoma expansion. Early aggressive physiotherapy should be avoided; however, assisted ROM exercises may begin 48–72 h after trauma, within a pain-free arc, progressing to isometric, isotonic, and dynamic exercises as tolerated. Large symptomatic haematomas may benefit from aspiration. Non-steroidal anti-inflammatory drugs and corticosteroids are useful for the treatment of flare-ups [2,20,21] (Figure 3D–F).

### 8.2. Surgical Treatment

Surgical excision is reserved for symptomatic lesions failing conservative management, with indications including intractable pain from mechanical irritation, neurovascular compression, or functional limitation. Historically, surgery was delayed 6–18 months after injury to minimize recurrence risk; however, recent evidence suggests early excision carries minimal recurrence risk when maturity is confirmed. Current practice recommends individualized timing based on aetiology: 6 months for traumatic MO, 12 months for spinal cord injury, and 18 months for traumatic brain injury. Marginal excision is generally adequate, though recurrence has been reported [22,23,24].

## 9. Imaging Follow-Up

Imaging follow-up should be tailored to the stage of disease evolution. In early lesions, short-interval follow-up (3–4 weeks) with radiography or CT may confirm the development of peripheral ossification [2,7]. During the intermediate phase, imaging performed at approximately 6–8 weeks may demonstrate maturation of the characteristic zonal pattern [2]. Once the lesion reaches the mature phase and symptoms improve, further imaging is generally unnecessary unless clinical progression raises concern for an alternative diagnosis [2,8].

## 10. Conclusions

Myositis ossificans is a benign, self-limiting soft-tissue disorder whose radiologic appearance may closely mimic aggressive soft-tissue neoplasms. The main source of diagnostic error lies in the interpretation of imaging findings without adequate consideration of lesion evolution. A step-by-step radiologic assessment, integrating clinical context with the time-dependent evolution of imaging features, is therefore essential to achieve an accurate diagnosis. Awareness of stage-specific findings, especially at MRI, allows radiologists to recognize myositis ossificans with confidence and to distinguish it from malignant entities. By emphasizing key imaging hallmarks, common interpretative pitfalls, and the complementary role of different imaging modalities, this review highlights the pivotal role of radiology in guiding appropriate patient management. Correct imaging interpretation may prevent unnecessary biopsies or surgical interventions and supports a conservative approach in most cases, with imaging follow-up tailored to disease evolution. A stage-based imaging approach may serve as a practical framework for the evaluation of suspected myositis ossificans, facilitating accurate diagnosis and appropriate clinical management.

## Figures and Tables

**Table 1:** Summary table for muscle oedema patterns, common causes, and associated imaging features. Modified from McMahon et al. [17].

Distribution Pattern	Main Causes	Key Imaging Clues
Diffuse	Inflammatory myopathies (polymyositis, dermatomyositis)	Proximal distribution, lower limbs more affected
	Inclusion body myositis	Distal muscle distribution
	Viral myositis	Associated systemic viral symptoms
	Drug-related myopathy	History of medication use (e.g., statins, zidovudine)
Focal	Muscle strain	Involvement at musculotendinous junction
	Contusion	Located at muscle–bone interface
	Laceration	Linear intramuscular defect
	Pyomyositis	Rim-enhancing intramuscular abscess
	Diabetic muscle infarction	Non-enhancing muscle with perifascial fluid
	Compartment syndrome	Confined to a single muscle compartment
	Denervation	Distribution following a specific nerve
	Tumor	Focal enhancing mass
Multifocal	Delayed onset muscle soreness	Follows pattern of muscle overuse
	Pyomyositis	Multiple abscesses
	Sarcoid myopathy	Multiple nodular lesions
	Metastases	Multiple enhancing masses

**Table 2:** Summary table highlighting key diagnostic features and common differentials of MO.

Tips and Tricks for Differential Diagnosis
A history of trauma, with symptom improvement and functional recovery after 8 weeks—even in the absence of medical treatment—should point towards a self-limiting process such as MO.
Non-neoplastic muscle oedema preserves muscle fibers architecture (striated/checkerboard pattern) and regresses over time, unlike neoplastic muscle oedema, which is infiltrative, causes disruption of muscle fibres, and increases progressively.
The growth of the MO mass/lesion during the intermediate and mature phases consistently follows the long axis of the affected muscle.
The location of soft-tissue calcifications within the mass/lesion (central or peripheral), their temporal evolution (zone phenomenon), and their relationship with the adjacent cortical bone allow a reliable differential diagnosis from soft-tissue and osseous lesions.

## Data Availability

No new data were created or analyzed in this study.
